# Smoking-attributable mortality among Korean adults in 2019

**DOI:** 10.4178/epih.e2024011

**Published:** 2023-12-19

**Authors:** Yeun Soo Yang, Keum Ji Jung, Heejin Kimm, Sunmi Lee, Sun Ha Jee

**Affiliations:** 1 Department of Epidemiology and Health Promotion, Institute for Health Promotion, Graduate School of Public Health Yonsei University, Seoul, Korea; 2 Health Insurance Policy Research Institute, National Health Insurance Service, Wonju, Korea

**Keywords:** Smoking, Population attributable risk, Mortality, Tobacco

## Abstract

**OBJECTIVES:**

Tobacco use ranks among the leading preventable causes of death worldwide. This study was conducted to calculate the mortality rate attributable to smoking in Korea for 2019 and to highlight the importance of tracking and monitoring smoking-related deaths for public health purposes.

**METHODS:**

Population attributable risk (PAR) was used to estimate the number of deaths related to smoking in 2019. PAR percentages were applied to the estimated mortality figures for various diseases, with PAR determined based on relative risk (RR). Levin’s formula was used to calculate PAR, and RR was adjusted for age and alcohol consumption using Cox proportional hazards regression model to derive disease-specific regression coefficients. The analysis incorporated previously determined smoking rates from 1985, and use rates of novel tobacco products were not considered.

**RESULTS:**

The findings revealed a total of 67,982 smoking-attributable deaths in Korea in 2019, 56,993 of which occurred in men and 11,049 in women. The PAR of smoking for various causes of death in adult men was highest for lung cancer at 74.9%, followed by pneumonia (29.4%), ischemic heart disease (42.3%), and stroke (30.2%). For women, the PAR for smoking-related death was highest for lung cancer (19.9%), followed by stroke (7.6%), pneumonia (5.7%), and ischemic heart disease (9.1%).

**CONCLUSIONS:**

In countries experiencing rapid fluctuations in smoking rates, including Korea, regular studies on smoking-related mortality is imperative. Furthermore, it is necessary to investigate smoking-related deaths, including the prevalence of novel tobacco product use, to accurately gauge the risks associated with emerging tobacco products.

## GRAPHICAL ABSTRACT

[Fig f2-epih-46-e2024011]

## Key Message

This study analyzed deaths attributable to smoking in Korea in 2019, revealing that a total of 67,982 individuals lost their lives due to smoking. Among these, 56,993 were men and 11,049 were women, with the highest smoking-related mortality rate observed in men due to lung cancer at 74.9%, and in women due to lung cancer at 19.9%. Through these findings, this research emphasizes the importance of tracking and monitoring smoking-related deaths for public health.

## INTRODUCTION

Tobacco use ranks among the leading preventable causes of death worldwide. The World Health Organization (WHO) reports that tobacco use is responsible for approximately 7 million deaths each year, with the majority of these attributable to direct use [1]. Despite clear evidence of the health risks linked to tobacco use, the prevalence of smoking remains high in regions around the world, and smoking-related deaths continue to rise. In the United States alone, smoking causes over 480,000 fatalities annually, including deaths from lung cancer, heart disease, stroke, and chronic obstructive pulmonary disease (COPD) [2]. Efforts to reduce smoking rates have been successful in some countries; earlier research conducted a comparative assessment of smoking rates, exploring the correlation between each country’s smoking prevalence and its tobacco control strategies. The findings revealed that countries with the lowest smoking rates had placed the greatest emphasis on national tobacco control policies [3,4].

Numerous studies have demonstrated a direct correlation between tobacco use and elevated risk of premature mortality. For instance, a large-scale prospective study conducted in the United States revealed that smokers faced a markedly greater risk of mortality from all causes, including lung cancer, cardiovascular disease, and respiratory disease, compared to non-smokers [5]. A European study indicated that between 30% to 60% of excess mortality was attributable to smoking, with disproportionately high rates observed for lower socioeconomic groups [6]. Moreover, the detrimental effects of tobacco use extend beyond individual health outcomes to include societal and economic consequences. Smoking-related healthcare costs place a considerable burden on healthcare systems, and reduced productivity resulting from smoking-related illnesses and premature deaths can profoundly impact economic growth and development [7].

Tobacco use not only presents a serious threat to public health but also has a substantial economic impact. The WHO estimates that the global economic burden of tobacco use exceeds US$1 trillion annually. This figure includes both direct healthcare costs and productivity losses resulting from premature death and disability [4]. Furthermore, the burden of tobacco use disproportionately affects low-income and middle-income countries, where the prevalence of smoking is often higher and healthcare resources may be limited [4].

Tobacco use is a leading cause of death worldwide. In 2019, tobacco smoking was reportedly responsible for approximately 7.69 million (95% uncertainty interval [UI], 7.16 to 8.20) deaths and 200 million (95% UI, 185 to 214) disability-adjusted life years globally, and it was the leading cause of death among men (accounting for 20.2% [95% UI, 19.3 to 21.1] of deaths in this group). Of 769 million tobacco-related fatalities, 668 million (86.9%) occurred among current smokers [8]. In a study that estimated the population attributable fraction (PAF) by calculating relative risk (RR) in the Korean population for ever smoking and in the Asian population for passive smoking, it was found that in 2009 in Korea, tobacco smoking was responsible for 20,239 (20.9%) incident cancer cases and 14,377 (32.9%) cancer deaths among adult men, as well as 1,930 (2.1%) cancer cases and 1,351 (5.2%) cancer deaths among adult women [9]. Another study revealed that the PAFs attributable to combined smoking and alcohol consumption, as well as those attributable to each factor separately, were higher for cancer mortality than for cancer incidence [10]. In Korea, smoking was linked to over 48,000 deaths in 2019, representing 22.7% of all deaths that year [11]. In 2014, the U.S. Department of Health and Human Services emphasized the importance of tracking smoking-related fatalities for informing public health policies, allocating resources, and increasing public awareness of the dangers of smoking. Similarly, the WHO in 2021 stressed the importance of monitoring smoking-related deaths to evaluate the effectiveness of smoking control measures and to examine progress toward global health goals [12,13].

Finally, a report of the surgeon general [14] contended that smoking should be a key focus when addressing health issues across all United States. They emphasized the importance of tracking and monitoring smoking-related deaths in developing improved strategies to address related problems. Notably, smokers typically face a life expectancy that is at least 10 years shorter than those who have never smoked [15].

This study incorporated mortality data up to the year 2019 and serves as a follow-up to research published in 2013 [16]. The study objective was to estimate the all-cause and disease-specific mortality risks associated with current smoking in Korea, as well as to determine the population-attributable risk (AR) fraction, by using large-scale cohort data from Korea.

## MATERIALS AND METHODS

### Study population

We used data from 3 large cohort studies: The National Health Insurance Service-Korean Cancer Prevention Study (NHIS-KCPS), the Korean Metabolic Syndrome Mortality Study (KMSMS), and the Korean Cancer Prevention Study-II (KCPS-II).

#### NHIS-KCPS

In 2019, the Korean Cancer Prevention Study (KCPS) underwent a name change to become the NHIS-KCPS. This cohort is composed of insured government employees and private school staff who were enrolled in the Korean Medical Insurance Corporation (now known as the National Health Insurance Service) through the Government Employees’ Union and Private School Staff Union. All participants underwent at least 1 routine physical examination between 1992 and 1999 [17,18]. We investigated smoking history from 1992 to 1995 and analyzed causes of mortality up to 2019. Of the 2,384,045 individuals in the cohort, those younger than 30 years old or with incomplete data on smoking status were excluded from the analysis. The final number of study participants was 1,327,003. The mean± standard deviation age was 45.3± 11.1 years for men and 49.6± 12.2 years for women. Among the men participants, 58.5% were current smokers, while 93.5% of women participants had never smoked (Table 1).

#### KMSMS

The KMSMS is a retrospective cohort study based on information from private health examinations conducted between 1994 and 2004 across 18 centers in Korea [19]. Mortality data were linked using official personal identification numbers. The study initially identified 560,566 participants aged 20 years or older who had undergone a baseline health assessment. From this group, 52,548 individuals were excluded due to being younger than 30 years old. An additional 203,994 participants were excluded because information on their smoking status was missing. Consequently, the final cohort comprised 304,007 individuals, consisting of 173,091 men and 130,916 women. The mean age was 46.9± 11.7 years for men participants and 47.0± 10.0 years for women participants. Among men, 51.5% were current smokers, while 89.4% of women had never smoked (Table 1).

#### KCPS-II

The KCPS-II is a prospective cohort study utilizing data from private health examinations conducted between 1994 and 2013 across 18 Korean centers [20]. Of the 270,514 individuals enrolled, 25,372 were excluded due to being younger than 30 years; additionally, 18,184 participants were excluded because information was unavailable regarding their smoking status. Consequently, the final number of study participants was 226,958 (134,633 men and 92,325 women). The mean age was 44.2± 10.0 years for men and 45.4± 10.6 years for women. Among men participants, 45.6% were current smokers, whereas 90.5% of women participants were never-smokers (Table 1).

### Data collection

Participants were instructed to self-report several lifestyle factors, which included smoking status (never, former, or current smoker), alcohol consumption (measured in grams of ethanol per day), engagement in exercise (yes or no), and medical history, comprising hypertension (yes or no), diabetes (yes or no). Height and weight were measured directly, with participants dressed in light clothing and without shoes. Body mass index (BMI) was calculated as weight in kilograms divided by the square of height in meters. We used the WHO Asian-Pacific classification system to categorize BMI: those with a BMI below 18.5 kg/m^2^ were considered underweight, while those with a BMI ranging from 18.5 kg/m^2^ to 22.9 kg/m^2^ were classified as normal weight. Above this range, classifications of “overweight” (23.0≤ BMI< 25.0 kg/m^2^) and “obese” (BMI≥ 25.0 kg/m^2^) were applied [20,21]. Blood pressure measurements were taken with the participants seated, using either a standard mercury sphygmomanometer or an automatic manometer [17,20,22].

The study outcomes included both cancer-related mortality and overall mortality. Mortality status, including the date and cause of death, was ascertained by cross-referencing with records from the National Statistical Office up to the year 2019. The causes of death and overall mortality rates for each disease and cancer type were determined using death certificates obtained from the National Statistical Office. Data abstractors then classified the causes of cancer-related deaths and overall mortality according to the International Classification of Diseases, 10th edition (ICD-10). The follow-up period for all-cause mortality began on the date of participant enrollment and concluded at the time of death. For disease-specific mortality, follow-up also began at enrollment but ended at the date of death attributable to a disease as defined by the corresponding ICD-10 code.

### Statistical analysis

#### Smoking-related disease risk

The NHIS-KCPS cohort data used in this study have limited representativeness because the participants consisted of government employees, private school staff, and their dependents. Consequently, data from KMSMS and KCPS-II participants were also used. In this study, the RR was calculated using data from NHIS-KCPS, KMSMS, and KCPS-II in a complementary manner.

Using non-smokers as a reference group, we calculated the RR of overall mortality associated with current smoking, as well as mortality risks for specific diseases, while adjusting for age and alcohol consumption in both genders. We then applied a meta-analysis approach to combine the calculated RRs of smoking from each of the 3 cohorts. Meta-analysis is a research method employed to systematically synthesize or amalgamate the results from separate, independent studies, using statistical techniques to determine an overall or “absolute” effect [23].

Weights were applied in the synthesis of research findings. These weights were determined as follows.

Additionally, the heterogeneity of the integrated data was evaluated using the Higgins I^2^ value (I^2^ = [Q−df]/Q× 100%) and the Q test (Q statistic). Even when research results exhibit heterogeneity, the Q statistic may not indicate significant findings at the typical significance level of less than 0.05. Therefore, following Fleiss’s recommendation, a significance level of 0.1 was adopted, with values below 0.1 indicating heterogeneity rather than homogeneity. The I^2^ statistic represents the proportion of total variation attributable to heterogeneity across study results. An I^2^ value of less than 25% is considered to indicate low statistical heterogeneity, values between 25% and 75% suggest moderate heterogeneity, and values exceeding 75% indicate high heterogeneity [24]. Based on this, a model was chosen with a threshold I^2^ value of 50%. When the I^2^ value was below 50%, a fixed-effects model was employed, while for I^2^ values of 50% or higher, a random-effects model was applied. In this study, a variance components model was used in a conservative approach. All analyses were conducted using SAS version 9.4 (SAS Institute Inc., Cary, NC, USA) and R version 3.4 (R Foundation for Statistical Computing, Vienna, Austria).

#### Smoking rates

The Korea Tuberculosis Association and Gallup Korea have conducted surveys to assess historical smoking rates in Korea. The surveys carried out by the Korea Tuberculosis Association in 1980, 1985, and 1990 are regarded as representative studies of adult smoking rates [25,26]. According to the 1980 smoking survey, the rates of smoking among men and women over 30 years old were 79.4% and 12.5%, respectively [27]. The 1985 survey reported smoking rates of 71.1% for men and 10.9% for women [28], while the 1990 survey found that 68.2% of men and 6.7% of women were smokers at the age of 30 years. While a steady decline in smoking rates has been observed among men over the past 20 years, the rates among women have remained relatively unchanged [29]. However, these surveys did not account for several new tobacco products, which have seen growing popularity. Moreover, given that the health risks associated with tobacco use can persist for many years, if not decades, the smoking rates from 1985 were used in the present study [29-31]. Additionally, we incorporated the smoking rates of 65.6% for men and 6.4% for women from the 1995 National Health and Health Behavior Survey, conducted by the Korea Institute for Health and Social Affairs. These rates were employed by the Korea Centers for Disease Control and Prevention (KCDC; currently the Korea Disease Control and Prevention Agency) to estimate smoking-ARs in 2019.

#### PAR and smoking-related deaths

Population attributable risk (PAR) was calculated using Levin’s formula [32]. RR was determined using the Cox proportional hazards regression model and disease-specific regression coefficients. These RR estimates were obtained from values generated via the Cox proportional hazards model, adjusted for age and alcohol consumption status. The AR was then computed based on the RR values that were previously calculated.

Attributable risk= smoking rate in 1985× (relative risk−1)/[smoking rate in 1985× (relative risk−1)+1]

The PAR for overall mortality was calculated first, followed by a corresponding calculation for each disease. Calculations were performed for a total of 41 diseases, yielding values for 38 diseases in men and 40 in women. As previously noted, the smoking prevalence figures used to calculate the PAR were obtained from the 1985 National Survey on Smoking Rates, which reported rates of 71.1% for men and 10.0% for women. The number of deaths attributable to smoking was estimated by multiplying the total number of deaths reported by the National Statistical Office of Korea in 2019 by the PAR for overall mortality and for each specific disease [33].

In the comprehensive analysis, models were adjusted: initially for age only, then for both age and alcohol consumption status, and finally for age, alcohol consumption, physical activity, and BMI [30]. To maintain consistency with previous research [16] and with findings from the KCDC [34], we present the model that was adjusted for age and alcohol consumption status.

### Ethics statement

The Institutional Review Board of Yonsei University granted approval for these studies (NHIS-KCPS IRB No. 4-2001-0029, KCPS-II IRB No. 4-2011-0277, KMSMS IRB No. 4-2007-0065).

## RESULTS

In 2019, a total of 295,110 fatalities were reported to the National Statistical Office, comprising 160,322 men and 134,788 women. Among individuals over 30 years old, there were 290,451 deaths, including 157,479 men and 132,975 women. Table 1 compares the general characteristics of the population cohort used in this study. The NHIS-KCPS enrolled participants from 1992 to 1999, the KMSMS from 1996 to 2004, and the KCPS-II from 1994 to 2013.

Disparities in participant characteristics were noted among the 3 cohorts. The NHIS-KCPS cohort comprises public officials, private school teachers, and their dependents. The KMSMS cohort is drawn from individuals attending 18 comprehensive examination centers nationwide, while the KCPS-II cohort consists of participants from 18 centers within Korea. Furthermore, differences were observed in average age, smoking rates, and alcohol consumption rates of men and women across the 3 cohorts.

Table 2 presents the smoking-related RRs of specific diseases and overall mortality in men, as observed in the NHIS-KCPS, KMSMS, and KCPS-II cohort studies. As previously detailed, the combined RRs were derived through a meta-analysis that integrated the RRs calculated from the NHIS-KCPS, KCPS-II, and KMSMS studies.

In the KMSMS and KCPS-II, the RRs of current smoking for all-cause mortality were 1.84 and 1.98, respectively. In contrast, the NHIS-KCPS displayed a RR of 1.58. A meta-analysis of these studies indicated a combined RR of 1.78, which was statistically significant. After categorizing the causes of mortality into all cancers, circulatory diseases, and other diseases, the RR for all cancers was found to be 2.07. This exceeded the risk for circulatory diseases, which was 1.50. Among all cancers, laryngeal and lung cancers—both closely linked to smoking—exhibited the highest RRs at 5.38 and 5.13, respectively. Furthermore, the RRs of mortality from esophageal cancer, COPD, atherosclerosis, oropharyngeal cancer, and bladder cancer were 3.39, 4.16, 2.82, 2.01, and 2.07, respectively.

For all fatalities among women, the RR was 2.01 in the KMSMS, 1.52 in the NHIS-KCPS, and 2.04 in the KCPS-II. A meta-analysis of these studies indicated a combined RR of 1.83, which was statistically significant. After categorizing the causes of death into all cancers, circulatory diseases, and other diseases, the RR for circulatory diseases was 1.81, surpassing that of all cancers (1.63). The RR of overall mortality was nearly identical between men and women; however, the risk varied for specific diseases. Laryngeal cancer presented the highest RR for women at 7.31, followed by lung cancer at 3.23, COPD at 5.66, and atherosclerosis at 3.40, as shown in Table 3.

Table 4 presents the PAR and the number of smoking-related fatalities derived from the 1985 smoking rates. Based on these rates of 71.1% for men and 10.7% for women, 67,982 fatalities were attributable to smoking: 56,993 among men and 11,049 among women. For men, lung cancer was the predominant cause of smoking-related mortality, accounting for 10,258 fatalities, followed by pneumonia with 3,598, ischemic heart disease with 3,246, and stroke with 3,198. In women, lung cancer also led to the highest number of smoking-related deaths at 969, followed by stroke with 830 deaths, pneumonia with 638, and ischemic heart disease with 546.

**Figure 1.**
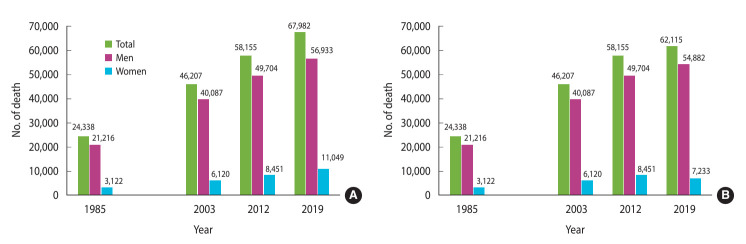
Trend of smoking attributable deaths in Korean men and women. Population attributable risks for 2019 were estimated using prevalence of smoking in (A) 1985 and (B) 1995.

Figure 1A illustrates the trends in smoking-related mortality for the years 1985 [28], 2003 [27], 2012 [16], and 2019. The number of smoking-related deaths steadily increased over these years.

In 2019, the number of smoking-related fatalities rose to 1.1 times the value recorded in 2012 for men and to 1.3 times the value for women. When compared to the figures from 1985, the number of smoking-related fatalities in 2019 had increased by a factor of 2.7 for men and 3.5 for women.

## DISCUSSION

Tracking and monitoring deaths related to direct smoking is essential in public health, as it yields critical insights for informing public health policy decisions and resource allocation. It also raises public awareness of smoking-related issues, helps assess the effectiveness of smoking control measures, facilitates monitoring of progress toward global health goals, and provides motivation for smoking cessation. In this study, we estimated the number of smoking-related deaths in Korea by analyzing long-term follow-up data from the KMSMS and NHIS-KCPS cohort studies. The findings indicate that 67,982 smoking-related deaths occurred in Korea in 2019, comprising 56,933 men and 11,049 women. Lung cancer accounted for the greatest number of smoking-related deaths (10,258 men and 969 women), followed by pneumonia, ischemic heart disease, and stroke.

This study updated the estimated number of fatalities attributable to smoking in Korea, employing the same smoking rates as the related study published in 2013 [16]. The increase in smoking-related deaths, despite a recent decrease in smoking prevalence, can be attributed to the fact that the population-level health risks associated with tobacco use persist for a minimum of several years and can last decades [29]. Regarding lung cancer, an increase in smoking prevalence has been observed to align with an increase in lung cancer incidence, typically with a 25-year lag [35].

Over the past 40 years, the smoking rate among Korean adult men has declined from its peak of 79.3% in 1980. In 1985, the estimated smoking-attributable mortality rates for adult men and women were 16.6% and 3.5%, respectively. In 2003, the ARs for smoking were estimated to be 30.8% for men and 5.7% for women, which increased to 34.7% for men and 7.2% for women by 2012. The ARs in 2019 were 36.2% for men and 8.3% for women, figures that align with those reported in previous research [16]. The KCDC indicated that in 2019, the ARs for men and women were 32.3% and 5.3%, respectively, with smoking responsible for 58,036 deaths (50,942 men and 7,094 women). We calculated the RR using smoking rates from 1995 and a meta-analysis of 4 datasets. According to a 1995 smoking survey, among adults at least 30 years old, the smoking rate was 65.6% for men and 6.4% for women [35]. Applying these 1995 rates to the present study, the total number of smoking-related deaths can be estimated at 62,115 (54,882 men and 7,233 women); additionally, the smoking-ARs were 34.9% for men and 5.4% for women, displaying no statistically significant difference relative to other studies (Table 5).

The estimated number of smoking-related deaths varies greatly depending on whether calculations are based on past smoking rates. When estimating fatalities attributable to smoking, methodological considerations must be considered, particularly given that Korea has experienced a steady decline in smoking rates since 1980.

Since the first survey in Korea in the 1980s, smoking rates among men have consistently declined, whereas rates among women fell from the 1980s to the mid-1990s, then rose, and have since stabilized at between 6% and 7%. However, the smoking rates reported to date have predominantly pertained to conventional cigarettes. With the emergence of various novel tobacco products, such as eliquid tobacco (for which research began in 2013), heated tobacco products (introduced to Korea in 2017), and JUUL (launched in Korea in May 2019), the prevalence of these products is on the rise. Although the cigarette smoking rate has been steadily decreasing since 1990, with the rate among adult men declining to 31.3% in 2021, smoking statistics must be developed to reflect the growing population of e-cigarette users. In response to these changes, key national surveys in Korea are undergoing revisions and expansions, including the National Health and Nutrition Examination Survey and the Youth Online Health Behavior Survey.

In a recent meta-analysis incorporating cohort data from 6 Asian countries, it was confirmed that the smoking rate among men has declined over time. Concurrently, the risk of death from all causes has risen among smokers. These findings suggest that smoking will persist as a key public health issue in Asian nations in the coming decades [36].

### Strengths and limitations

The strengths of this cohort study are noteworthy. First, the analysis utilized a large and nationwide sample. Second, it included long-term follow-up data that spanned more than 20 years. Finally, the tracking of disease identification was nearly perfect, with the exception of data pertaining to immigrants. However, the study also presents several limitations. First, the survey assessing smoking rates was conducted only once, at the beginning of the cohort study. Because smoking habits can change over time, relying on a single measurement to assess the effects of smoking may not provide a comprehensive understanding. Second, the follow-up duration in this study might have been insufficient to fully observe the impact of smoking [37,38]. Prior research suggests that the effects of smoking are more evident when monitored over a period of 40 years, as opposed to 20 years. Third, the smoking rate reported in this study reflects only the use of conventional cigarettes and does not account for emerging tobacco products. The study also did not consider the potential influence of passive smoking on smoking-related deaths, as accurately assessing this impact with the available data proved challenging. Fourth, the exclusive participation of Koreans, which may limit the applicability of the findings to other racial or ethnic groups. Fifth, the associations observed in this study may have been distorted by differences in risk factors such as diet, physical activity, and medical service utilization between smokers and non-smokers. Finally, the possibility of distortion in the results is dependent on the cause of death under investigation. Nevertheless, previous research on the relationship between smoking and mortality has indicated that adjusting for population characteristics and behavioral factors has a negligible impact on risk estimates [39]. In conclusion, in this study, we estimated the smoking rate, the number of smokers, and the number of smoking-related fatalities in Korea in 2019 and compared these figures to historical data to identify long-term trends. Despite a consistent decrease in smoking prevalence in Korea throughout the 1990s, the number of smoking-related deaths rose in the years 1985, 2003, 2012, and 2019. The findings of this study indicate that 67,982 deaths were attributable to smoking in 2019.

To understand the long-term health risks of smoking in countries experiencing rapid changes in smoking rates, including Korea, the number of smoking-related deaths should be regularly investigated. Furthermore, future research on the number of smoking-related deaths should consider the prevalence of emerging tobacco products to assess the risks posed by these new products.

## Figures and Tables

**Figure f2-epih-46-e2024011:**
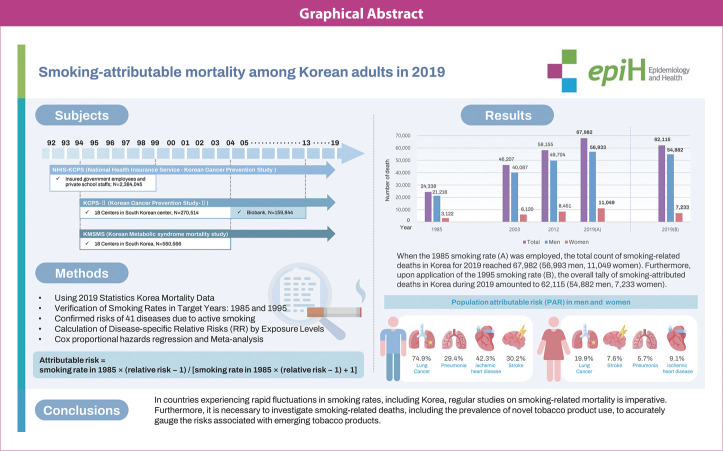


**Table 1.** General characteristics of included cohorts

Characteristics	NHIS-KCPS			KMSMS			KCPS-II		
	Men (n=845,932)	Women (n=481,071)	Total (n=1,327,003)	Men (n=173,089)	Women (n=130,914)	Total (n=304,003)	Men (n=134,633)	Women (n=92,325)	Total (n=226,958)
Enrollment years	1992-1999	1992-1999		1996-2004	1996-2004		1994-2013	1994-2013	
Follow-up duration, mean±SD (yr)	24.2±6.3	24.3±5.5		17.9±3.6	18.2±3.2		14.1±4.0	14.9±4.1	
Age, mean±SD (yr)	45.3±11.1	49.6±12.1	46.9±11.7	47.0±10.0	47.7±10.4	47.3±10.2	44.2±10.0	45.4±10.6	44.7±10.2
30-39	37.9	20.3	31.5	25.6	26.2	25.9	39.1	36.4	38.0
40-49	27.1	30.9	28.5	37.7	31.4	35.0	33.6	29.9	32.1
50-59	23.9	27.0	25.0	23.8	27.5	25.4	18.2	21.9	19.7
60-69	8.2	15.5	10.9	11.0	12.9	11.8	7.6	10.0	8.6
70+	2.9	6.3	4.1	1.9	1.9	1.9	1.5	1.8	1.6
BMI (kg/m^2^)									
<18.5	2.8	4.1	3.3	2.4	4.0	3.1	1.8	5.7	3.4
18.5-22.9	45.8	46.1	45.9	33.6	45.6	38.7	29.4	49.5	37.6
23.0-25.0	28.0	23.1	26.2	29.2	23.4	26.7	28.8	21.2	25.7
≥25.0	23.4	26.7	24.6	34.9	27.0	31.5	40.0	23.5	33.3
Alcohol consumption									
Yes	72.0	12.4	50.4	84.0	33.3	62.1	84.2	38.5	65.6
No	28.0	87.6	49.6	15.5	64.8	36.7	13.6	56.8	31.1
Missing	0.0	0.0	0.0	0.5	1.9	1.1	2.3	4.8	3.3
Smoking status									
Never smoker	20.8	93.5	47.1	21.4	89.4	50.7	26.4	90.5	52.4
Former smoker	20.8	2.2	14.0	27.1	5.3	17.7	28.1	5.3	18.8
Current smoker	58.5	4.3	38.9	51.5	5.3	31.6	45.6	4.2	28.7
Current smoker (n)	494,719	20,840	515,559	89,167	6,961	96,128	61,329	3,894	65,223
Amount smoked (cigarette/day)									
<10	3.2	19.5	3.8	9.9	34.3	11.7	13.0	40.4	14.6
10-20	66.7	69.5	66.9	31.5	34.2	31.7	39.4	33.5	39.1
>20	30.1	11.0	29.3	54.3	20.6	51.9	45.7	16.2	43.9
Missing	-	-	-	4.2	10.9	4.7	2.0	9.9	2.4
Smoking duration (yr)									
<5	3.0	14.0	3.4	8.8	15.7	9.3	1.8	7.7	2.1
5-9	6.5	10.5	6.6	1.3	11.6	2.1	4.7	17.7	5.4
10-19	30.9	13.6	30.2	22.6	31.8	23.2	39.5	40.4	39.5
20-29	23.1	16.3	22.8	29.3	15.3	28.3	32.7	16.1	31.6
≥30	15.8	24.0	16.2	19.5	5.8	18.5	16.9	5.9	16.2
Missing	20.8	21.7	20.8	18.5	19.8	18.6	4.7	12.2	5.1

Values are presented as %. NHIS-KCPS, National Health Insurance Service-Korean Cancer Prevention Study; KMSMS, Korean Metabolic Syndrome Mortality Study; KCPS-II, Korean Cancer Prevention Study-II; SD, standard deviation; BMI, body mass index.

**Table 2.** Relative risk of mortality from various diseases associated with current smoker status in Korean men

Cause of death	ICD-10 code	NHIS-KCPS (n=845,932)	KMSMS (n=173,089)	KCPS-II (n=134,633)	Combined cohort (n=1,153,654)
All-cause		1.58 (1.56, 1.60)	1.84 (1.76, 1.91)	1.98 (1.85, 2.11)	1.78 (1.55, 2.05)^1^
All cancers		1.83 (1.80, 1.87)	2.14 (2.00, 2.27)	2.32 (2.09, 2.56)	2.07 (1.80, 2.39)^1^
Oropharynx	C00-C14	2.01 (1.69, 2.38)	2.55 (1.47, 4.42)	2.18 (0.91, 5.18)	2.01 (1.69, 2.38)^1^
Esophagus	C15	2.77 (2.42, 3.18)	5.82 (3.21, 10.55)	2.73 (1.35, 5.52)	3.39 (2.14, 5.36)^1^
Stomach	C16	1.67 (1.59, 1.75)	1.75 (1.46, 2.11)	2.15 (1.59, 2.89)	1.68 (1.60, 1.76)^1^
Small intestine	C17	1.57 (1.11, 2.22)	3.67 (1.06, 12.74)	NE	1.67 (1.19, 2.33)^1^
Colon	C18	1.14 (1.06, 1.24)	1.38 (1.03, 1.86)	1.52 (0.94, 2.43)	1.17 (1.08, 1.26)^1^
Rectum	C19, C20	1.27 (1.15, 1.40)	1.39 (0.96, 2.01)	1.54 (0.85, 2.80)	1.28 (1.17, 1.41)^1^
Liver	C22	1.44 (1.37, 1.50)	1.70 (1.48, 1.96)	2.19 (1.72, 2.78)	1.70 (1.38, 2.10)^1^
Gallbladder	C23, C24	1.36 (1.25, 1.49)	1.14 (0.87, 1.49)	1.31 (0.87, 1.98)	1.34 (1.24, 1.45)^1^
Pancreas	C25	1.41 (1.31, 1.52)	1.67 (1.36, 2.04)	1.58 (1.17, 2.13)	1.45 (1.35, 1.55)^1^
Larynx	C32	5.29 (3.85, 7.25)	8.35 (1.97, 35.42)	4.85 (1.00, 23.64)	5.38 (3.97, 7.28)^1^
Lung	C34	4.76 (4.51, 5.03)	5.67 (4.84, 6.64)	5.39 (4.24, 6.85)	5.13 (4.52, 5.83)^1^
Brain	C71	0.93 (0.79, 1.10)	1.87 (1.14, 3.06)	2.59 (1.11, 6.06)	1.03 (0.88, 1.20)^1^
Thyroid	C73	1.45 (0.99, 2.08)	NE	1.04 (0.16, 6.69)	1.26 (0.90, 1.77)^1^
Leukemia	C91-C95	1.25 (1.09, 1.43)	1.32 (0.92, 1.90)	1.20 (0.71, 2.04)	1.25 (1.11, 1.42)^1^
Bladder	C67	2.09 (1.79, 2.44)	1.75 (1.12, 2.75)	3.50 (1.37, 8.95)	2.07 (1.79, 2.40)^1^
Kidney	C64	1.30 (1.11, 1.53)	0.97 (0.61, 1.55)	0.68 (0.30, 1.53)	1.24 (1.06, 1.44)^1^
Prostate	C61	NE	1.18 (0.86, 1.62)	1.99 (1.08, 3.65)	1.17 (0.86, 1.60)^1^
Circulatory		1.48 (1.44, 1.52)	1.77 (1.60, 1.97)	1.66 (1.43, 1.92)	1.50 (1.47, 1.54)^1^
Hypertensive disease	I10-I13	1.52 (1.37, 1.69)	1.16 (0.68, 2.01)	1.10 (0.45, 2.69)	1.50 (1.35, 1.66)^1^
Ischemic heart disease	I20-I25	1.74 (1.65, 1.83)	2.13 (1.78, 2.56)	2.40 (1.80, 3.21)	2.00 (1.65, 2.44)^1^
Arrhythmia	I47-I49	1.17 (0.98, 1.39)	1.40 (0.70, 2.80)	1.14 (0.43, 3.03)	1.18 (1.00, 1.39)^1^
Heart failure	I50	1.30 (1.15, 1.47)	1.25 (0.83, 1.90)	1.00 (0.46, 2.20)	1.29 (1.15, 1.45)^1^
Stroke	I60-I69	1.35 (1.30, 1.40)	1.67 (1.44, 1.93)	2.01 (1.55, 2.59)	1.61 (1.29, 2.01)^1^
Atherosclerosis	I70-I74	2.91 (2.39, 3.54)	2.24 (1.32, 3.79)	2.88 (1.18, 7.00)	2.82 (2.35, 3.37)^1^
Others					
Diabetes mellitus	E10-E14	1.43 (1.35, 1.52)	1.83 (1.44, 2.34)	2.54 (1.61, 4.02)	1.76 (1.32, 2.35)^1^
Mental disorders	F00-F09	1.22 (1.07, 1.38)	NE	NE	1.22 (1.07, 1.38)^2^
Sudden death	R96	1.48 (1.21, 1.82)	0.87 (0.45, 1.67)	0.85 (0.33, 2.20)	1.38 (1.14, 1.68)^1^
Aging	R54	1.39 (1.31, 1.48)	1.75 (1.29, 2.39)	1.17 (0.75, 1.82)	1.40 (1.32, 1.48)^1^
COPD	J44	2.69 (2.45, 2.95)	5.07 (3.48, 7.39)	6.28 (3.23, 12.2)	4.16 (2.36, 7.34)^1^
Tuberculosis	A15-A19	1.53 (1.34, 1.73)	NE	NE	1.53 (1.34, 1.73)^2^
Pneumonia	J09-J18	1.47 (1.38, 1.56)	1.87 (1.50, 2.34)	1.44 (0.98, 2.10)	1.57 (1.33, 1.86)^1^
Ulcer	K25-K27	1.83 (1.38, 2.41)	1.74 (0.61, 5.01)	3.02 (0.33, 28.12)	1.83 (1.40, 2.39)^1^
Liver cirrhosis	K74	1.40 (1.29, 1.52)	1.58 (1.06, 2.38)	1.80 (0.86, 3.76)	1.41 (1.30, 1.52)^1^
Accident	V01-V99	1.09 (1.03, 1.16)	1.34 (1.20, 1.50)	1.55 (1.30, 1.84)	1.30 (1.06, 1.58)^1^
Poisoning	X40-X49	1.87 (1.31, 2.65)	NE	NE	1.87 (1.31, 2.65)^2^
Unspecified cause	X58-X59	1.36 (1.14, 1.62)	NE	NE	1.36 (1.14, 1.62)^2^
Suicide	X60-X84	1.36 (1.27, 1.45)	NE	NE	1.36 (1.27, 1.45)^2^
Homicide	X85-Y09	1.38 (0.95, 2.02)	NE	NE	1.38 (0.94, 2.02)^2^
Injury, undetermined	Y10-Y34	1.34 (1.16, 1.54)	NE	NE	1.34 (1.16, 1.54)^2^

Values are presented as relative risk (95% confidence interval). ICD-10, International Classification of Diseases, 10th edition; NHIS-KCPS, National Health Insurance Service-Korean Cancer Prevention Study; KMSMS, Korean Metabolic Syndrome Mortality Study; KCPS-II, Korean Cancer Prevention Study-II; NE, not estimated due to small sample; COPD, chronic obstructive pulmonary disease.

1 Combined results via meta-analysis.

2 Results from National Health Insurance Service-Korean Cancer Prevention Study.

**Table 3.** Relative risk of mortality from various diseases associated with current smoker status in Korean women

Cause of death	ICD-10 code	NHIS-KCPS (n=481,071)	KMSMS (n=130,914)	KCPS-II (n=92,325)	Combined cohort (n=704,310)
All-cause		1.52 (1.49, 1.55)	2.01 (1.85, 2.18)	2.04 (1.78, 2.35)	1.83 (1.46, 2.29)^1^
All cancers		1.46 (1.40, 1.53)	1.75 (1.52, 2.01)	1.81 (1.44, 2.28)	1.63 (1.40, 1.90)^1^
Oropharynx	C00-C14	1.21 (0.69, 2.13)	1.76 (0.41, 7.62)	NE	1.21 (0.69, 2.13)^1^
Esophagus	C15	1.83 (1.06, 3.18)	8.75 (0.73, 104.99)	NE	2.45 (0.74, 8.11)^1^
Stomach	C16	1.16 (1.03, 1.30)	1.88 (1.20, 2.94)	1.61 (0.74, 3.50)	1.41 (0.99, 2.02)^1^
Small intestine	C17	NE	NE	NE	NE
Colon	C18	1.12 (0.95, 1.33)	1.16 (0.59, -2.29)	1.27 (0.46, 3.51)	1.13 (0.96, 1.33)^1^
Rectum	C19, C20	1.14 (0.91, 1.43)	1.03 (0.42, 2.57)	0.94 (0.23, 3.94)	1.13 (0.91, 1.40)^1^
Liver	C22	1.18 (1.02, 1.37)	1.60 (1.03, 2.48)	1.86 (0.89, 3.85)	1.24 (1.08, 1.42)^1^
Gallbladder	C23, C24	1.14 (0.97, 1.34)	0.97 (0.49, 1.91)	3.02 (1.48, 6.14)	1.41 (0.81, 2.48)^1^
Pancreas	C25	1.16 (0.99, 1.37)	1.17 (0.71, 1.92)	1.82 (0.79, 4.23)	1.18 (1.01, 1.37)^1^
Larynx	C32	7.31 (3.91, 13.66)	NE	NE	7.31 (3.91, 13.66)^2^
Lung	C34	3.18 (2.91, 3.47)	3.92 (3.02, 5.08)	2.92 (1.82, 4.69)	3.23 (2.98, 3.51)^1^
Brain	C71	NE	NE	NE	NE
Thyroid	C73	NE	NE	NE	NE
Leukemia	C91-C95	NE	NE	NE	NE
Bladder	C67	1.59 (1.12, 2.28)	NE	NE	1.59 (1.12, 2.28)^2^
Kidney	C64	NE	NE	NE	NE
Breast	C50	0.80 (0.57, 1.13)	1.47 (0.86, 2.52)	1.74 (0.84, 3.62)	1.19 (0.71, 1.98)^1^
Cervix	C53	1.68 (1.27, 2.23)	1.31 (0.40, 4.30)	NE	1.66 (1.26, 2.18)^1^
Ovary	C56	1.26 (0.92, 1.73)	1.41 (0.71, 2.80)	NE	1.28 (0.96, 1.71)^1^
Circulatory		1.54 (1.48, 1.60)	2.18 (1.80, 2.65)	1.85 (1.12, 3.08)	1.81 (1.36, 2.41)^1^
Hypertensive disease	I10-I13	1.38 (1.24, 1.54)	1.68 (0.66, 4.24)	1.29 (0.17, 9.74)	1.39 (1.24, 1.55)^1^
Ischemic heart disease	I20-I25	1.91 (1.76, 2.06)	2.39 (1.67, 3.41)	2.60 (1.41, 4.77)	1.93 (1.79, 2.08)^1^
Arrhythmia	I47-I49	1.51 (1.19, 1.91)	0.42 (0.06, 3.11)	NE	1.48 (1.17, 1.88)^1^
Heart failure	I50	1.52 (1.33, 1.72)	1.89 (0.94, 3.80)	1.57 (0.37, 6.71)	1.53 (1.35, 1.73)^1^
Stroke	I60-I69	1.43 (1.36, 1.50)	2.16 (1.66, 2.79)	1.92 (1.19, 3.09)	1.76 (1.27, 2.42)^1^
Atherosclerosis	I70-I74	2.14 (1.66, 2.76)	5.04 (2.50, 10.16)	5.13 (1.64, 16.07)	3.40 (1.71, 6.78)^1^
Others					
Diabetes mellitus	E10-E14	1.50 (1.37, 1.64)	2.18 (1.37, 3.48)	2.49 (1.20, 5.18)	1.79 (1.29, 2.48)^1^
Mental disorders	F00-F09	1.31 (1.16, 1.48)	NE	NE	1.31 (1.16, 1.48)^2^
Sudden death	R96	1.55 (1.03, 2.33)	2.59 (0.76, 8.86)	2.60 (0.32, 20.90)	1.66 (1.14, 2.43)^1^
Aging	R54	1.45 (1.37, 1.53)	1.92 (1.21, 3.07)	1.79 (0.81, 3.96)	1.45 (1.38, 1.54)^1^
COPD	J44	3.24 (2.82, 3.73)	7.76 (3.93, 15.30)	11.15 (3.11, 39.93)	5.66 (2.55, 12.56)^1^
Tuberculosis	A15-A19	1.42 (1.11, 1.83)	NE	NE	1.42 (1.11, 1.83)^2^
Pneumonia	J09-J18	1.53 (1.39, 1.68)	2.07 (1.33, 3.23)	2.23 (1.01, 4.92)	1.55 (1.42, 1.70)^1^
Ulcer	K25-K27	2.50 (1.74, 3.59)	4.35 (0.46, 41.36)	17.00 (0.86, 336.48)	2.60 (1.82, 3.71)^1^
Liver cirrhosis	K74	1.31 (1.05, 1.64)	2.64 (1.35, 5.17)	2.15 (0.65, 7.13)	1.74 (1.04, 2.91)^1^
Accident	V01-V99	1.18 (0.98, 1.41)	2.42 (1.91, 3.07)	2.73 (1.87, 3.98)	1.95 (1.11, 3.44)^1^
Poisoning	X40-X49	2.17 (1.11, 4.23)	NE	NE	2.17 (1.11, 4.23)^2^
Unspecified cause	X58-X59	1.39 (1.10, 1.75)	NE	NE	1.39 (1.10, 1.75)^2^
Suicide	X60-X84	1.57 (1.31, 1.89)	NE	NE	1.57 (1.30, 1.89)^2^
Homicide	X85-Y09	2.34 (1.02, 5.37)	NE	NE	2.34 (1.02, 5.37)^2^
Injury, undetermined	Y10-Y34	1.11 (0.80, 1.50)	NE	NE	1.11 (0.81, 1.52)^2^

Values are presented as relative risk (95% confidence interval). ICD-10, International Classification of Diseases, 10th edition; NHIS-KCPS, National Health Insurance Service-Korean Cancer Prevention Study; KMSMS, Korean Metabolic Syndrome Mortality Study; KCPS-II, Korean Cancer Prevention Study-II; NE, not estimated due to small sample; COPD, chronic obstructive pulmonary disease.

1 Combined results via meta-analysis.

2 Results from National Health Insurance Service-Korean Cancer Prevention Study.

**Table 4.** PAR^1^ from smoking and smoking-attributable death in Korean men and women in 2019

Variables	ICD-10 code	Men		Women		Total
		Total PAR	Smoking-attributable death	PAR	Smoking-attributable death	
All-cause		36.2	56,933	8.3	11,049	67,982
All cancers		43.8	21,875	6.4	1,971	23,846
Oropharynx	C00-C14	42.2	418	2.1	6	424
Esophagus	C15	63.3	902	15.5	20	922
Stomach	C16	32.9	1,625	4.2	112	1,737
Small intestine	C17	33.4	61	-	-	61
Colon	C18	11.3	321	1.6	41	362
Rectum	C19, C20	17.0	369	1.5	19	388
Liver	C22	34.0	2,646	2.7	75	2,721
Gallbladder	C23, C24	19.7	507	4.2	101	608
Pancreas	C25	24.1	826	1.7	52	878
Larynx	C32	76.2	220	40.5	12	232
Lung	C34	74.9	10,258	19.9	969	11,227
Brain	C71	4.6	31	1.7	10	41
Thyroid	C73	13.1	14	3.8	10	24
Leukemia	C91-C95	15.8	166	-	-	166
Bladder	C67	44.3	517	6.1	23	540
Kidney	C64	13.8	92	-	-	92
Prostate	C61	10.4	213	-	-	213
Breast	C50	-	-	2.1	55	55
Cervix	C53	-	-	6.7	60	60
Ovary	C56	-	-	3.0	37	37
Circulatory		30.9	205	8.1	26	231
Hypertensive disease	I10-I13	25.9	465	4.3	166	631
Ischemic heart disease	I20-I25	42.3	3,246	9.1	546	3,792
Arrhythmia	I47-I49	12.1	101	5.0	57	158
Heart failure	I50	17.0	384	5.5	245	629
Stroke	I60-I69	30.2	3,198	7.6	830	4,028
Atherosclerosis	I70-I74	56.9	463	20.7	145	608
Others		-	-	-	-	-
Diabetes mellitus	E10-E14	35.3	1,447	8.0	317	1,764
Mental disorders	F00-F09	12.7	153	3.4	82	235
Sudden death	R96	20.2	99	6.6	26	125
Aging	R54	22.3	937	4.8	449	1,386
COPD	J44	69.8	2,236	33.4	398	2,634
Tuberculosis	A15-A19	28.2	275	4.7	30	305
Pneumonia	J09-J18	29.4	3,598	5.7	638	4,236
Ulcer	K25-K27	37.4	100	14.8	31	131
Liver cirrhosis	K74	22.8	284	7.5	66	350
Accident	V01-V99	16.6	412	9.4	93	505
Poisoning	X40-X49	37.7	48	11.6	7	55
Unspecified cause	X58-X59	20.5	112	4.1	33	145
Suicide	X60-X84	20.6	1,790	6.1	201	1,991
Homicide	X85-Y09	20.6	30	13.3	20	50
Injury, undetermined	Y10-Y34	19.4	208	1.3	7	215

PAR, population attributable risk; ICD-10, International Classification of Diseases, 10th edition; COPD, chronic obstructive pulmonary disease.

1 PARs for 2019 were estimated using prevalence of smoking in 1985.

**Table 5.** Smoking-attributable risks of mortality in a literature review and the present study

Genders	Meng (1985) [28]	Jee et al. (1981-2003) [27]	Jung et al. (2012) [16]	Present study (2019)^1^	Present study (2019)^2^	KCDC (2019) [34]
Men	16.6	30.8	34.7	36.2	34.9	32.3
Women	3.5	5.7	7.2	8.3	5.4	5.3

KCDC, Korea Centers for Disease Control and Prevention; PAR, population attributable risk.

1 PARs for 2019 were estimated using the prevalence of smoking in 1985.

2 PARs for 2019 were estimated using the prevalence of smoking in 1995.
